# Prevalence of anxiety towards COVID-19 and its associated factors among healthcare workers in a Hospital of Ethiopia

**DOI:** 10.1371/journal.pone.0243022

**Published:** 2020-12-08

**Authors:** Simegnew Kibret, Diriba Teshome, Efrem Fenta, Metages Hunie, Tadese Tamire

**Affiliations:** Department of Anesthesia, Debre Tabor College of Health Sciences, Debre Tabor University, Debre Tabor, Ethiopia; Chinese Academy of Medical Sciences and Peking Union Medical College, CHINA

## Abstract

**Background:**

The World Health Organization declared the outbreak of COVID-19 as a pandemic on 11 March 2020. Healthcare workers are directly involved in the prevention, diagnosis, treatment, and care of patients with COVID-19.This study aims to assess the prevalence of anxiety and its associated factors towards the COVID-19 outbreak among healthcare workers in a Hospital of Ethiopia.

**Methods:**

A Hospital-based survey study was conducted on a total of 305 Healthcare workers in a Hospital of Ethiopia. Bivariable and multivariable logistic regression were used to analyze data between independent variables with anxiety. Variables with a p-value of <0.2 were transformed into multivariate analysis. Crude and adjusted odds ratios with 95% CI, p-values of <0.05 were used to show the strength of association and level of significance.

**Results:**

The prevalence of CVID-19 anxiety was 63%. In multivariate logistic regression, age of 30–39 (AOR, 3.05; 95% CI, (1.70, 5.47) and age of ≥40 (AOR, 11.32; 95% CI (3.37, 37.98), being married (AOR, 3.56; 95% CI, (2.30, 6.38), having chronic illness (AOR, 3.43; 95% CI, (1.59,7.43), having suspected COVID-19 family members (AOR, 5.20; 95% CI, (2.11, 12.78), and not having an access to PPEs (AOR, 2.55; 95% CI, (1.43, 4.56) were statistically significantly associated with anxiety.

**Conclusion:**

Being married, having a chronic illness, having suspected COVID-19 family members, not having access to PPEs, and age greater than or equal to 30 years were identified as risk factors for anxiety of Healthcare Workers towards COVID-19.

## Introduction

COVID-19 first reported on December 30, 2019, from Wuhan, China [1] and the World Health Organization declared the outbreak a Public Health Emergency of International Concern on 30 January 2020 and a pandemic on 11 March 2020 [2].The common symptoms of the disease are fever, cough, fatigue, myalgia, and extrapulmonary manifestations [3–7]. The disease has an incubation period of 3–6 days while it needs 5–6 days to be manifested and hospitalization is expected to be within 7–8 days [4, 5, 8].

As per the WHO report on 30^th^ September 2020, there have been about 33,722,075 cases and 1,009,270 deaths of COVID-19. There are 1,182,927cases and 25,881 deaths in Africa; and 74,584 cases and 1,191deaths in Ethiopia [9]. However, there might be underreporting of the number of cases due to diagnostic insufficiency, low testing capacity, and fragile health care system, especially in developing countries.

The American psychological association defines anxiety as an emotion characterized by feelings of tension, worried thoughts, and physical changes like increased heart rate [10].

Healthcare workers are directly involved in the prevention, diagnosis, treatment, and care of patients with COVID-19 who might beat high risk of developing anxiety. The ever-increasing number of confirmed and suspected cases, high workload, depletion of personal protection equipment, lack of specific drugs, increased risk of infection for families and colleague, feelings of being inadequately supported, and unable to adhere to prevention strategies may all contribute to this mental burden of the HCWs [11–13]. Healthcare workers perceive a greater risk of anxiety due to their exposure to the patients who are most poorly which adds further stress. This problem may be compounded in countries with under invested health sectors due to their underline poverty. Ethiopia is largely sharing this burden among other sub-Saharan countries [13–18].

After the COVID-19 pandemic, outbreak prevention and control measure like quarantining, closing and suspension of transportations, avoiding public gatherings, and even refraining from different important public and family events may rescue the general population and healthcare workers from anxiety due to reduced autonomy and issues like income, job, security and safety issues [19, 20].

If anxiety left untreated, it may have long-term health effects on healthcare workers and affect them to properly discharge their responsibilities including combating the COVID-19 outbreak. Furthermore, it may burden the under-resourced and fragile health sector by the cost of treatment and minimizing effective manpower resources [21, 22]. This study might add information for the scientific world and might be baseline data for further researches in Ethiopia.

The aim of this research to assess the prevalence of anxiety towards COVID-19 and its associated factors among HCWs in a Hospital of Ethiopia.

## Methods and materials

### Study period and setting

The study was conducted in Debre Tabor General Hospital from May 15 to June 15, 2020. The ethical clearance was obtained from Ethical Review Committee of Debre Tabor University College of Health Sciences Research and Community Service Coordination Office on May 11/ 2020 with protocol number Ref CHS/1089/2020. Letter of permission to conduct was obtained from, Debre Tabor General Hospital administrative body. Informed written consent was secured from every study participant before the start of the interview after telling them about the objective of the study. Confidentiality and anonymity were ensured. The hospital is found in the South Gondar Zone, which is one of the Zones found in the Amhara regional state. Debre Tabor is found 669km North West of Addis Ababa, the capacity of Ethiopia. The Hospital has about 350 HCWs.

### Study design

Hospital-based; survey-based cross-sectional study design was employed.

#### Source population

All healthcare workers working in Debre Tabor General Hospital.

#### Study population

Healthcare workers who are participated in actual data collection during the study period working in Debre Tabor General Hospital.

### Sampling size and technique

The sample size was not predetermined, and all available healthcare workers from May 15 to June 15 were surveyed.

### Study variables

#### Dependent variable

Anxiety (Yes/No)

#### Independent variables

Socio-demographic variables like age, sex, marital status, religion, educational level, work experience, chronic illness, suspected COVID-19 family member, accessibility of PPEs, Having family member, Participation in the previous outbreak, and monthly income.

### Data collection technique

Data were collected via a structured questionnaire. We used the English version of the GAD-7 scale to measure anxiety. TheGAD-7 scale was reliable with Cronbach alpha of 0.92 and test re-test reliability (intraclass correlation = 0.83) with good validity [23]. It is calculated by assigning scores 0, 1, 2, 3 for not at all, several days, more than half of days, and nearly every day responses for how often have you been bothered the seven listed symptoms of anxiety over the last 2 weeks. Adding the scores, the GAD-7 scale ranging from 0 to 21. The total scores of measurements were interpreted as normal (0–4), mild (5–9), moderate (10–14), and severe (15–21). The cut-off score for detecting anxiety is ≥ 5.This scale was reliable and valid Data was collected by assigned health care professionals (data collectors).

### Data quality assurance

A pretest was done on 5% of the study participants at Felege Hiwot Referral Hospital in Bahir Dar. After training was given for data collectors, data was collected and properly filled on the prepared questionnaire.

### Data entry and analysis

The data was coded and entered into SPSS version 23.Independent variables were analyzed by using binary logistic regression with anxiety (dependent variable) and those with a p-value of ≤0.2fromthe bivariate analysis were fitted to a multivariate logistic analysis to check their association with the outcome variable. Odds ratios with 95% CI (strength of association) and p-value were computed to identify associated factors. Tables were used to present data and summarized with frequency and percentage. P-values of <0.05 were considered as statistically significant.

## Ethics statement

The ethical clearance was obtained from Ethical Review Committee of Debre Tabor University College of Health Sciences Research and Community Service Coordination Office on May 11/ 2020 with protocol number Ref CHS/1089/2020. Letter of permission to conduct was obtained from, Debre Tabor General Hospital administrative body. Informed written consent was secured from every study participant before the start of the interview after telling them about the objective of the study. Confidentiality and anonymity were ensured.

### Operational definitions

#### Anxiety

an emotion characterized by the feeling of tension, worried thoughts, and physical changes like increased heart rate.

#### Anxiety scale

Anxiety (defined as a total score of ≥5 in the Generalized Anxiety Disorder-7) was assessed by a seven-item, GAD-7scale [23], which is defined as follows;

The seven items will be scored as follows: Not at all = 0, Several Days = 1; More than half the days = 2; Nearly every day = 3, then by adding the scores, the total scores of measurements will be interpreted as normal (0–4), mild (5–9), moderate (10–14), and severe (15–21).

Over the last 2 weeks, how often have you been bothered by the following problems?

1. Feeling nervous, anxious, or on edge
2. Not being able to stop or control worrying
3. Worrying too much about different things
4. Trouble relaxing
5. Being so restless that it is hard to sit still
6. Becoming easily annoyed or irritable
7. Feeling afraid as if something awful might happen

## Results

### Sociodemographic characteristics of healthcare workers

A total of 305 HCWs have participated with a response rate of 87%. Among the participant's females (65.9%) from sex, age group 20–29 (48.9%) from age in years, and orthodox Christian (92.8) from religion were the dominant study characteristics in our study. Most of our study participants are BSc degree holders, while most of them have work experience of 1–5 years. The majority of the respondents are unmarried, do not have a chronic illness, do have participated in the previous outbreak, and do not have access to PPEs (Table 1).

**Table 1:** Sociodemographic characteristics of HCWs at a Hospital of Ethiopia.

Variables	Frequency	Percentage
**Age (years)**		
20–29	149	48.9
30–39	121	39.7
≥ 40	35	11.5
**Sex**		
Male	104	34.1
Female	201	65.9
**Marital status**		
Unmarried	158	51.8
Married	147	48.2
**Education level**		
Diploma	39	12.8
BSc Degree	225	73.8
MSc and above	41	13.4
**Having family members**		
Yes	211	69.2
No	94	30.8
**Religion**		
Orthodox	283	92.8
Protestant	12	3.9
Muslim	10	3.3
**Presence of chronic illness**		
Yes	66	21.6
No	239	78.5
**Previous outbreak participation**		
Yes	229	75.1
No	76	24.9
**Suspected COVID-19 family member**		
Yes	43	14.1
No	262	85.9
**Work experience in years**		
1–5	151	52.3
6–10	122	40.0
> 10	22	7.2
**Accessibility of PPEs**		
Yes	95	31.1
No	210	68.9
**Income in Ethiopian Birr**		
<3000	26	8.5
3000–6000	115	37.7
6000–9000	93	30.5
> 9000	71	23.3

### Prevalence and severity of anxiety towards COVID-19 among healthcare workers

The overall prevalence of anxiety among HCWs towards COVID-19 was about 63%. Being married (34.8%), having family members (42.3%), and not having access to PPEs (47.2%) were anxious (Table 2). Among HCWs 11.1% of them developed severe anxiety, while 26.2% were mild, and moderate 25.6%.

**Table 2:** Prevalence of anxiety towards COVID-19 among HCWs at a Hospital of Ethiopia.

Variables	Presence of Anxiety	
	Yes, N (%)	No, N (%)
**Age (years)**		
20–29	76 (24.9)	73 (23.9)
30–39	85 (27.9)	36 (11.8)
≥ 40	31 (10.2)	4 (1.3)
**Sex**		
Male	62 (20.3)	42 (13.8)
Female	130 (42.6)	71 (23.3)
**Marital status,**		
Unmarried	86 (28.2)	72 (23.6)
Married	106 (34.8)	41 (13.4)
**Education level**		
Diploma	26 (8.5)	13(4.3)
BSc Degree	134 (43.9)	91 (29.8)
MSc and above	32 (10.5)	9 (3.0)
**Family members**		
Yes	129 (42.3)	81 (26.6)
No	63 (20.7)	31 (10.2)
**Religion**		
Orthodox	185 (60.6)	98 (32.1)
Protestant	2 (0.7)	10 (3.3)
Muslim	5 (1.6)	5 (1.6)
**Presence of chronic illness**		
Yes	56 (18.4)	10 (3.3)
No	136 (44.6)	103 (33.8)
**Previous outbreak participation**		
Yes	145 (47.5)	84 (27.5)
No	47 (15.4)	29 (9.5)
**Suspected COVID-19 family member**		
Yes	35 (11.5)	8 (2.6)
No	157 (51.5)	105 (34.4)
**Work experience in years**		
1–5	96 (31.5)	65 (21.3)
6–10	78 (25.6)	44 (14.4)
> 10	18 (5.9)	4 (1.3)
**Accessibility of PPEs**		
Yes	48 (15.7)	47 (15.4)
No	144 (47.2)	66 (21.6)
**Income in Ethiopian Birr**		
<3000	16 (5.2)	10 (3.3)
3000–6000	71 (23.3)	44 (14.4)
6000–9000	54 (17.7)	39 (12.8)
> 9000	51 (16.7)	20 (6.6)

### Risk factors of COVID-19 anxiety among healthcare workers

In multivariate logistic analysis; age greater than 30 years old, being married, presence of chronic illness, having suspected COVID-19 family members, and lack of accessibility of PPEs were statistically significantly associated with anxiety (Table 3).

**Table 3:** Bivariate and multivariate analysis of factors associated with anxiety of COVID-19 among HCWs at a Hospital of Ethiopia.

Variables	Presence of anxiety		COR (95% CI)	AOR (95% CI)	P-value
	Yes	No			
**Age (years)**					
20–29	76	73	1	1	
30–39	85	36	2.27(1.34, 3.76)	3.05(1.70, 5.47)	< 0.0001
≥ 40	31	4	7.44 (2.50, 22.13)	11.32 (3.37, 37.98)	< 0.0001
**Marital status**					
Unmarried	86	72	1	1	
Married	106	41	2.16 (1.34, 3.49)	3.56 (2.30, 6.38)	< 0.0001
**Presence of chronic illness**					
Yes	56	10	4.24 (2.07, 8.71)	3.43 (1.59,7.43)	0.002
No	136	103	1	1	
**Suspected COVID-19 family member**					
Yes	35	8	2.93 (1.31, 6.56)	5.20 (2.11, 12.78)	< 0.0001
No	157	105	1	1	
**Accessibility of PPEs**					
Yes	48	47	1	1	
No	144	66	2.14 (1.30, 3.51)	2.55 (1.43, 4.56)	0.02

## Discussion

During the COVID-19 outbreak, the number of patients requiring treatment increases significantly, which strains the healthcare system [12] especially in developing countries like Ethiopia due to lack of resources for prevention, diagnosis, and treatment. Healthcare workers perceive a greater risk of anxiety due to their exposure to the patients who are most poorly which adds further stress [13, 15].

Research from previous epidemics/pandemics (such as the SARS outbreak 2003, the MERS epidemic 2012, or Ebola outbreaks in West Africa) shows that HCWs can experience a broad range of psychological morbidities including trauma, which might endure for many months after the outbreak [24].

The overall prevalence of COVID-19 anxiety among HCWs of in a Hospital of Ethiopia was found to be about 63%. A multicenter study done in China by Liu et al. on 1563 medical staff found the prevalence of anxiety (defined as a total score of ≥5 in the Generalized Anxiety Disorder-7) to be 44·7% [25], the study conducted in China by Wang C, et al. (53.8%) [26], Dai Y, et al. (39.1%) [27] and Lai J, et al. (44.6) [28]. The possible explanation might be due to poor healthcare facilities.

Our study revealed that age of 30–39 (AOR, 3.05; 95% CI, (1.70, 5.47)) and age of ≥40 (AOR, 11.32; 95% CI (3.37, 37.98)),being married (AOR, 3.56; 95% CI, (2.30, 6.38)), having chronic illness (AOR, 3.43; 95% CI, (1.59,7.43)), having suspected COVID-19 family members (AOR, 5.20; 95% CI, (2.11, 12.78)), and not having access to PPEs (AOR, 2.55; 95% CI, (1.43, 4.56))were statistically significantly associated with anxiety.

In line with our finding, a rapid review and meta-analysis of 59 papers by Kisely S, et al. in 2020 on the occurrence, prevention, and management of the psychological effects of emerging virus outbreaks on HCWs found that being younger, more junior, parents of dependent children, or having an infected family member were significantly associated with anxiety [29] and having an intermediate professional title were associated with anxiety (AOR, 1.82; 95% CI, 1.38–2.39), and healthcare workers engaged in the direct diagnosis, treatment, and care of patients with COVID-19 were associated with anxiety (AOR, 1.57; 95% CI, 1.22–2.02) [28].

## Conclusion

Being married, having a chronic illness, having suspected COVID-19 family members, not having access to PPEs, and the age group of 30–39 and greater or equal to 40 years might increase the risk of anxiety towards COVID-19.

## Recommendation

Based on our findings, we recommend Hospital to avail PPEs and insure proper utilization of it. We suggest Ethiopian Government to give more emphasis for HCWs having chronic illness, suspected COVID-19 family members and older age groups to minimize their anxiety.

## Supporting information

S1 Checklist (DOCX)

S1 File (SAV)

## Abbreviations

AOR: Adjusted Odds Ratio
CI: Confidence Interval
COR: Crude Odds Ratio
COVID-19: COVID-19 Disease
GAD: Generalized Anxiety Disorder
HCWs: Healthcare Workers
PPEs: Personnel Protective Equipments
WHO: World Health Organization
